# Interpretative Phenomenological Analysis for the Comparison of Polish and Foreign Dentistry Students’ Concerns during the Covid-19 Pandemic

**DOI:** 10.3390/healthcare9060765

**Published:** 2021-06-19

**Authors:** Agata Trzcionka, Irena Zalewska, Marta Tanasiewicz

**Affiliations:** Department of Conservative Dentistry with Endodontics, Faculty of Medical Sciences in Zabrze, Medical University of Silesia, Plac Akademicki 17, 41-902 Bytom, Poland; izalewska@sum.edu.pl (I.Z.); martatanasiewicz@sum.edu.pl (M.T.)

**Keywords:** COVID-19, pandemic, stress, quality of life, the interpretative phenomenological analysis

## Abstract

Introduction: At the beginning of 2020, the worldwide infection of coronavirus (SARS-CoV-2) changed many aspects of human lives. The controlled lockdown was not only an obligatory lifestyle change to communities across the globe, but it was also an emotional struggle. The aim of the presented study was to identify and compare the main difficulties that final-year students (both Polish and foreigners) of Dentistry, Faculty of Medical Sciences in Zabrze Medical University of Silesia had to cope with during the first wave of the pandemic. Application of the biographical method in the form of essays written was done. Authors discussed the following issues: possible losses and benefits subjectively felt by individuals due to the COVID-19 pandemic, adaptation to online type of studying, students’ opinions regarding change of habits, and psychological impact of the lockdown. Students’ responses varied between groups in the aspect of what they considered as the biggest struggle of the pandemic time period. Adaptation to e-learning was easier for the group of foreign students. The Polish group considered it as a serious stress factor. Foreigners were much more worried about not being able to help their families in comparison to Polish students. Polish students’ fear was related to the fear of virus transmission to their older relatives. Both groups were aware of how serious the situation was and of the possible impact of lack of practical classes on their future internships. As benefits of that situation, both groups listed development of new hobbies and increased sport activities. The Polish group emphasized improvement of interpersonal bonds during the lockdown in their families. The main conclusion proved an awareness of the seriousness of the situation in which students of medical, especially dental faculties found themselves. Concerns were related to the form of further studies and the possibility of taking up employment after graduation. Polish students declared more intense concerns about e-learning and remote study than foreign-language students who were more familiar with this form of communication. A particularly valuable form of teaching for students may be increased numbers of online consultations, since even though they cannot replace practical classes, they may still be helpful in explaining doubts and simply “being there”. Encouraging young people to discover constructive benefits of the pandemic can also be one of a task-oriented strategies of help.

## 1. Introduction

The first case of SARS-CoV-2 infection was confirmed in China in December 2019. Subsequently, patients with syndromes of high fever, dry cough, chest pain, and general fatigue were identified in many Chinese cities and provinces [1]. At the beginning of 2020, the world faced alarming information that coronavirus (SARS-CoV-2) infection was quickly spreading across the world. On March 11th, the World Health Organization (WHO) officially announced that the viral infection fulfilled the criteria to be classed as a worldwide pandemic. The number of patients with COVID-19 was rapidly increasing across all continents-except the Antarctic (by 20 April 2021, there were already 141,549,845 cases reported globally, 3,021,397 deaths). The situation induced the implementation of emergency protective measures by the governments of countries all over the world. In Poland the first case of SARS-CoV-2 infection was confirmed on 4 March 2020. Sixteen days later epidemic status was announced [2]. Decisions were quickly made and the closure of most of the public institutions, temples and also borders was announced. Most of the mass events were canceled as well.

Several studies indicated potential mental health consequences of facing lockdown, pandemic restriction, and obligatory lifestyle changes [3,4,5,6]. Increased stress levels, anxiety disorders, depression, and even post-traumatic stress disorder due to pandemic may constitute long-term psychological problems for. Osofsky et al. emphasized the differences between facing both past technological and natural disasters and the current pandemic. They indicated that social isolation hindering supportive influenced on relationships and unpredictability considering the duration of the crisis occurred as major psychological concerns. They also observed that separation from the family members in the situation of their severe illness appeared to be one of the most difficult experiences of that time [4].

Undoubtedly, the situation was an exceptional struggle for many students, as there was a sudden disturbance to their studies due to the closure of educational institutions. It might be especially difficult and stressful for the international students in their final year as not only were their studies interrupted, but they were also trapped in a foreign country due to the closure of the Polish borders. Imperative for rapid adaptation to new online form of classes, waiting for decisions about the new construction of exams must have been especially difficult [2,7,8]. Severe Acute Respiratory Syndrome (SARS) and the H1N1 flu pandemic also resulted in temporary separation of local and foreign students and employees from their usual studying routine or occupational activities [7,8,9,10].

The outbreak of the COVID-19 pandemic forced dental schools all over the world to change the methods of teaching and implement new techniques. As was discussed by Bennardo et al., this completely new situation will undoubtedly change the future approach to dental education, and it is very probable that the e-learning technique would be implemented [11]. In another work Bennardo also examined the awareness of COVID-19 among dental students in Italy. It was observed that future dentist needed to be more conscious about the risk of exposure to the Sars-CoV2 virus in day-to-day work [12]. Many other researchers also observed the need to educate not only dental students, but also dental practitioners on the COVID-19 pandemic [13,14,15,16].

To understand major concerns, fears, and possible benefits of the current crisis it is very important to look for a qualitative method which will give a chance to get an insight into human emotions. These requirements are fulfilled by the biographical method, widely used in sociological and educational studies. Creative forms of the biographical method include diaries, essays, narratives, letters, and records. This method is appreciated in many sociological and psychological studies as a valuable scientific tool for detecting a wide range of emotions [17,18,19,20]. Reilly and Silk pointed out that these forms were helpful in two aspects: to discover the history of the person and to analyze the impact of the experience on individuals’ emotions. They highlighted the usage of essays and narratives as an important tool in mutual understanding and creation of supportive attitude [19].

Our qualitative study applied the biographical method in the form of essays written by final-year dental students.

The aim of the study was to:

- identify and compare the main difficulties students had to cope with;
- analyze potential benefits and losses felt by each individual due to the pandemic and lockdown.

## 2. Materials and Methods

### 2.1. Participants

The study involved 40 (20 foreigners and 20 Polish) students in their fifth year of Dentistry at the Faculty of Medical Sciences in Zabrze, Medical University of Silesia in Katowice. The particular situation that students suddenly had to face was probably most stressful for those who were attending their last year of studies. The fifth year of studies is the one during which most practical classes take place, and it is obvious that for future dentists, practice is of special concern. Taking that specific situation into account, the authors decided to concentrate on that particular group of students.

- Inclusion criteria: Fifth year dentistry students who gave their written consent to take part in the study, who had online classes and stayed in Poland.
- Exclusion criteria: Students who did not agree to take part in the study, of different years than fifth, in case of foreigners—those who left Poland before the borders were closed.
- Final criteria: Students who discussed in their essays 4 aspects chosen by the senior researcher (adaptation to e-learning, closure of the borders, social distancing, and risk of infection).

The consent for conducting the research was obtained from the Bioethical Committee.

### 2.2. Essays

Students were asked to write essays covering topics attached to a given framework. This preliminary framework was created by the authors to facilitate understanding of the initial concept of the study. 

Time allocated for writing essays was after the first wave of the COVID-19 pandemic ended. The form of essays enabled the participants to describe their feelings, emotions, and experiences in their own way. The given topic of the essay was very general: “Being a student during COVID-19 pandemic”. It gave the participants a possibility to choose what the most important issue they had to deal with was. They were invited to express their opinion on the influence of the pandemic on each aspect of their life, not only the one strictly connected to academic life.

### 2.3. Data Analysis

According to Creswell (1998) and Morse’s (1994) recommendations, it was decided to include in the study a small sample. Cited authors pointed out that small size makes it possible to analyze the data in detail. Szymaniak claimed that unlike other methods, the biographical method did not require a large statistical sample to be valid. Even single unit’s personal dramas, actions and dilemmas may constitute important information helpful in creating more general sociological conclusions [20].

This claim also corresponds with the results presented by Dobosz and Gierczyk in their review considering the application of biographical method in the social sciences. The authors selected 30 studies from the literature for further analysis. Sample sizes of selected articles varied from 1 to 89 and the wide range of topics included the impact of the disease to the mental health, transboundary education, geographical migration, and effectiveness of educational programs [21]. Roberts pointed out that sometimes even a single individual may be a representative of the larger group [22]. There were a number of qualitative studies reflecting a picture of social history in Sars-CoV-2 disaster using smaller samples. Kendall-Thackett noticed that even the group of narratives not exactly matched to the usual requirement for papers could form a separate unique quality helpful in obtaining a better sense of the current challenge [23]. In the research by Morgan et al. with the usage of the biographical method, the total sample consisted of 11 respondents [24]. According to Creswell, smaller sizes in narrative studies and case histories were sufficient [25]. Another limitation of the research may be a lack of male members in the Polish group. Our study was based on the students that volunteered to participate in it. Women represented 75% of the whole sample of participating students. This result corresponds with a literature review by Dobosz and Gierczyk. In their analysis of 30 studies with application of a biographical narrative, women constituted 80% of all respondents [21].

The Interpretative Phenomenological Analysis (IPA) was used to analyze the data. IPA enables the detailed examination of single human-being experiences. As described by Smith et al. this research tool has three underpinnings: philosophical approach, interpretative endeavor, and being idiographic in its commitment to analyze each case separately [26].

After the analysis of essays, the authors redefined the criteria of the study, with the supervising senior researcher having the greatest experience in conducting qualitative research. For the analysis to be more trustworthy, a second author coded the manuscripts. Polish students have been marked as P while English Division as E. Each student was given a number (1–20).

The supervisor did not have classes with the students participating in the study. On the basis of essay analysis, it was discovered that the most frequent and important aspects in the essays were:

- adaptation to e-learning;
- closure of the boards;
- social distancing;
- risk of infection.

After analysis of obtained the essays, the researchers decided that chosen aspect would be classified as important if it occurred in at least two essays in each group.

## 3. Results

The English Division students were composed of 20 students in the age range of 24–28. They came from the United Kingdom, Iraq, Sweden, Saudi Arabia, and Taiwan. Polish students that took part in the research were ages 24–27.

Discussion of chosen aspects by the students is presented in Table 1 and Table 2.

All respondents declared that the necessity to attend online courses was a challenge. They all agreed that this kind of education has both advantages and disadvantages. For Polish students, the most stressful were technical issues related to the e-learning platform and with the quality of internet connection but also the loss of practical classes, which they took as a cause of lack of self-assurance in future clinical work. Saving time by staying home and no need to travel to the university was the main advantage of the e-learning mentioned by both foreigners and Polish students. However, they were worried about the lack of practical classes and its impact on their future skills (Table 2). Both groups pointed out that the hybrid technique of learning could be implemented in the future. They found it crucial to have all theoretical classes in online version.

The information about the closure of Polish borders surprised both groups of students. It would seem that it would be more difficult to be accepted by the foreigners. However, most of them highlighted that since they took a decision about studying abroad, they live in a kind of lockdown, so that decision taken by the Polish government did not change their lives very much. They were much more scared that it will influence their future career as most of them planned to have their postgraduate internship in their countries. The problem of border closure affected one of the Polish students whose father worked abroad (Table 2). For most of the Polish students the closure of the borders was problematic only because traveling was impossible (f.e. “…I had do reerange my holiday plans…”, “I was planning a trip around the Europe and it had to be cancelled.”).

The analysis of the students’ essays proved that all respondents from both groups had to face similar problems because of social distancing. They missed meetings with their friends and had a lack of activity due to the closure of gyms. Even though it was a difficult time for most of us, future dentists pointed that pandemic gave them a chance for self-development, new hobbies, getting new skills and improvement of others. Polish students mentioned the possibility of strengthening ties with their families (Table 1 and Table 2). It needs to be mentioned that one student from the English Division declared being diagnosed with depression due to isolation. That person declared to be under supervision of a psychiatrist.

There were only three students (all from the English Division) who mentioned the aspect of fear about their own health. In the essays of English Division students, the problem of the virus and the disease was mainly discussed (Table 1). All respondents in both groups of students emphasized the fear and worries about health of their relatives and friends, especially those staying abroad. They were concerned that in case of disease they cannot help their families. It is probably caused by the fact that as a medical student they are more aware than the rest of society. In Polish essays we could find concerns about being the source of the infection for relatives. We can assume that they realize how serious the situation was (Table 1 and Table 2).

## 4. Discussion

During the coronavirus pandemic, students were exposed to a significant number of stressful situations, such as disturbances in the course of academic classes, reduced social and family contacts, especially in the case of foreign-language students who have remained in the country where they study, away from their families, as in the case of the analyzed group [4,26,27]. Limiting academic classes for students of dentistry only to online theoretical classes may cause great concern about the specifics of the study program, which is largely based on practical clinical exercises. Pokhrel and Chhetri noticed that concentration of problems might appear in the case of year-end examination due to decreased number of classes and consultations in contact. That situation directly affected both groups of students participating in our study as they were all final-year students. In their review on impact of the pandemic on teaching and learning process Pokhrel and Chhetri pointed out that during the current crisis students faced the most serious disruption of education in history. They described adaptation to that “Education in emergency” as challenging for teachers and students [28]. Education-related challenges for medical and dental schools, as well as their affiliated hospitals, are significant. Reilly and Silk emphasized the importance of sharing individual experiences of medical workers in the time of pandemic and its educational and supportive role for the whole medical community [19]. Singh et al., who assessed attitudes of students towards interprofessional education and significance of this preparation during the pandemic proved that students from both clinical and non-clinical programs valued opportunities for learning together and felt that IPE enhances awareness of team members’ roles and responsibilities and improves collaboration [29]. It was reported that open communication among students, clinical teachers, and administrative staff would enhance mutual trust and facilitate adequate cooperation at a time of particular epidemic danger [30]. Batra et al. proved that dental students had sufficient knowledge regarding the COVID 19 pandemic but also showed that there is a need to restructure the educational curriculum to prepare students to handle COVID-19 and future pandemics [31]. Studies show pandemics, such as COVID-19 increased psychological stress, and the consequences of quarantine might lead to emotional disturbance, depression, irritability, insomnia, anger, and emotional exhaustion among other health and mental health conditions [4,6,8].

An additional distress, specific for students, may be the need for a quick adaptation to a variety of e-learning platforms and systems, self-motivation for online courses or accessibility of the internet [28,32,33]. Probably, the problems with web-based communication declared by the Polish students were the result of the fact that they did not use internet for online communication in their day-to-day life. While this was well known and eagerly done by the foreigners who used that means of communication with their families and friends before the pandemic was announced. In the English Division group, there was one student who was a blogger; undoubtedly, he was the one for whom it was the easiest to fully use internet and he offered his help to his friends. Both groups were equally scared of problems with the quality of the internet connection that they had no influence on.

For both groups of students, the health of their relatives and friends, especially those staying abroad, was very important. They were concerned that in case of disease they could not help their families. It might be caused by the fact that both groups of students had personal contact with the disease in their families. They experienced the risk of COVID-19, death of infected friends or observed their relatives suffering from it. Ossofsky emphasized that fear for relatives being ill was very strong di-stressor impacting mental health [4]. This claim was also confirmed by the study by Evans et al. [27]. Two students from the English Division described medical professionals in their families fighting with the disease on the front line-in England and Sweden, where, as we know, many people were suffering from COVID-19. Reilly and Silk emphasized that exchange of pandemic experiences among the health care community helped to fight challenges, understand the crisis, and strengthen solidarity in this group of professionals [19]. Information obtained from family members caused these students to be more aware of how the treatment and consequences of the disease looked. Surveys conducted among Chinese students indicated that the public perception of health risks among college students in China presented valuable information on their perception of health risks. Generally, the hazards that were perceived as posing the greatest health risk were those belonging to the social health risk category; items related to technology risks received the lowest percentage [31]. University students played a particularly important role in debates about voluntary quarantines for SARS. In response to an official quarantine of an entire residential building at the Central University of Finance and Economics (CUFE) on 16 April 2003, CUFE announced that the university would cancel all classes until May 8. It became the first university to cancel classes and to dismiss students to avoid cross-infection of SARS [9]. Rumors about the closure of all dormitories drove many students home. College students mobilized traditional values such as patriotism and collectivism to call for voluntary quarantines both during SARS in Beijing and at the beginning of H1N1 flu. As grass roots risk reduction efforts, voluntary quarantines aimed to cut off the transmission channel between epicenters and areas affected by epidemics through the use of travel restrictions and voluntary quarantines. Viewed as possible high-risk populations, university students from epicenters took the bottom-up initiative to impose voluntary quarantines in both epidemics, which appealed to the trope of responsible citizens [10,31].

The main differences in the perception of the situation by foreign-language students studying at the university was due to restrictions on mobility and possibility of coming back to their home countries. Similarly to Polish students, foreigners felt particular anxiety about their loved ones left behind in their home countries. A good point of reference for such results may be the situation described in the comparative analysis of the feelings of students from Russia and Belarus, in which different ways of managing the pandemic were taken [8]. Overall, students from universities in Belarus where the restrictions were less strict in the field of quarantine/self-isolation, reported more positive psycho-emotional conditions than those from Russia [8]. However, research on students and teachers carried out in one of the universities in Pakistan indicated that there was much greater awareness of the risk among the academics, which led to rationalization of the problem and had a positive impact on the emotional state even when isolated [34,35,36]. Attitudes to the pandemic and coping strategies in the current crisis showed a wide range of variation and may depend on natural resilience, mindfulness, and optimism [3,27]. Protective factors against fear and negative mood states were provided by benefits also observed in the time of Sars-Cov-2 [6,37]. In the study by Tang et al., 21.4% of respondents declared that their current life improved in the time of the pandemic. Chinese children and adolescents regarded the longer time spent with their families and opportunities for new hobbies as beneficial [21]. Evans et al., in their study conducted with an application of the biographical method, also emphasized improvement of family relation as one of the possible benefits, but also pointed out some incidents of interpersonal distress [27]. Strengthened family bonds during the pandemic was strongly highlighted by Polish students, spending lockdown mostly in their family homes [37]. Both groups of our participants regarded more time for new activities, like cooking or running as important beneficial effects, which, they hoped, would be a constant part of their lives even after the pandemic.

The pandemic has already shown significant psychological symptoms related to anxiety, stress, and depression in analyzed social groups [38]. Studies highlighted the unpredictability of pandemic time, stressors in different aspects of life and its impact on the mental health of the communities [3,4,27,28]. Longitudinal studies are necessary to analyze the long-term impact of this situation on the psychological state of the students and draw conclusions about the cause and effect between the variables involved [8,9].

During the current pandemic, Orgiles et al. analyzed and compared coping strategies used by adolescents and children selected from three different countries, Portugal, Italy, and Spain. Results varied from emotionally oriented coping style, combined with an increased level of fear and behavioral repercussions to avoidance-oriented or task-oriented strategies proving stronger adaptation abilities. The chosen methodological tool in their research was an online survey completed by the parents of children and adolescents in the age range of 3–18 [28]. Reilly and Silk emphasized that among a great number of quantitative data considering COVID-19 pandemic there was a constant need of sharing memories from the crisis selected and described by their own words [19]. A number of studies highlighted an increasing role of qualitative research methods in modern science [19,39,40].

The biographical method is used in psychological, sociological, and pedagogical sciences. Studies by Szymaniak or Kelterchsman highlighted the importance of “critical incidence” which usually also constitutes a “meaningful incidence”. Personal respondents’ descriptions of these incidents were useful in further analysis to get an insight into human concerns and emotions [20,39]. The COVID-19 pandemic fulfilled the definition of “critical incidence” or may be even considered as a “turning point” in many lives. Szymaniak pointed out that for comparison and explanation of multidimensional human experiences-quantitative methods might be insufficient [20]. Although highly valued, the biographical method is not free from limitations. Although the subjective character of biographical narrative is often considered by the quantitative researchers as a weakness of this method, there are also a lot of studies appreciating this tool for contextualization of historical and individual aspects [19,20,40,41]. Roberts described the debate in the literature considering validity and reliability of biographical studies and pointed out researchers recommending “a look beyond” standardized criteria [22]. These methods focus on the person as “creator of meanings”. Evans et al. in their research, showed the experimental approach prioritized respondent’s own meanings and interpretation. They used the inductive analysis as their research tool [22]. Roberts described an analytic induction as one of the bases for the further analysis of narratives [27].

According to this recommendation, authors of this study constructed the preliminary hypothesis of potential emotional and educational difficulties for Polish and foreign students during the coronavirus pandemic. The next step was comparing and searching for differences and similarities between particular cases. Huber and Hyde claimed that selection of the research method should be based not only on reliability or data but focusing on finding an answer to the research question [40]. Students’ essays presented a wide range of aspects. Originally there were no limitations considering the length of the narrative. For the comparative purposes, they were cut and extracted in fragments corresponding best to the framework questions. The Sars-Cov-2 pandemic had the hallmarks of the “turning point” of many lives, including young people in the beginning of their careers. Thakur emphasized from the students’ perspective the need for support programs and techniques for improving resilience. She highlighted that online counseling, volunteering, and mental health promotion may be a true support for the youth in current crisis [42].

The authors consider it as the main limitation of the presented study, because the context of individual cases, heartbreaking descriptions of emotional struggles or complicated family situation were much more clearly visible when analyzing the whole essay.

## 5. Conclusions

The analysis of students’ essays allowed for a cautious but adequate assessment of the moods prevailing among Polish and foreign-language students. The main conclusion proved an awareness of the seriousness of the situation in which students of medical, especially dental faculties found themselves. Concerns were related to the form of further studies and the possibility of taking up employment after graduation.

Polish students declared more intense concerns about e-learning and remote study than foreign-language students who were more familiar with this form of communication. As in other studies, the authors pointed out to the anxiety prevailing in the health and wellbeing of the family members—both those living nearby and those left behind in distant family countries. The need for further development of supportive strategies is emphasized in the literature [39,40,41].

A teaching form particularly valuable for students may be an increased number of online consultations, since even though they cannot replace practical classes, they may still be helpful in explaining doubts and simply “being there”. Encouraging young people to discover constructive benefits of the pandemic can also be one of a task-oriented strategies of help.

## Figures and Tables

**Table 1:** Extracts from the English Division students’ essays discussing chosen issues.

Aspect	Examples of Extracts	Participant	Number of Similar Opinions
daptation to e-learning	“Another great thing is that I’ve been saving over an hour a day in travel time which I’ve been able to utilise to more productive tasks…”; “…I 110% prefer the style of teaching we have had during this pandemic as opposed to the layout of seminars prior to the pandemic. They have been so much more beneficial. I feel like the webinar classes are a lot more interactive and more productive…”	E1	16
	“…school became tough due to poor internet connection, and I was forced to borrow a computer…”	E2	
	“…the internet is very poor here and I don’t have access to my laptop…”	E3	
	“…Losing your last and final semester in Dentistry was definitely a loss in my eyes the practical work missed…”	E4	
Closure of the boards	“…as soon as it was known that Poland was going into lockdown many international students that I know, rushed back to their home countries and told me to do the same but I was stubborn. What if it would prevent me from taking my diploma exams? I wasn’t willing to take the risk…”	E1	10
	“…but the main concern that really affected me mentally is the uncertainty of what is going to happen to you in regards to career your education that you have invested so much in. will it finish? How long are you stuck here?…”	E4	
	“…I now also face the issue of what my future is going to look like. I wish to return to England and start my career there…”	E5	
	“…The isolation itself isn’t too bad as I’ve lived in Poland for a long time…”, “…This is also affecting all of my UK internship possibilities–because obviously foreigners are not their main priority right now and with all the borders being closed…”	E3	
	“…The initial thing that transpired to me was that I had to leave Poland, and being in a dilemma of choosing whether to stay or leave was nerve-wracking. All this time, my thoughts rotated on the possibility of schools reopening and me not being able to go back…”	E2	
Social distancing	“…I hate running but I figured why not. It’s been 3 weeks and I have been running 5 km everyday…”; “…I started to bake cookies and cakes, I was experimenting more with my cooking…”; “…Human contact. I’d forgotten what that was. After that moment I knew that I needed to go outside daily for my own sanity.”	E1	8
	“…Unable to enjoy and do daily outdoor activities and be more interactive and the freedom of meeting people became dramatically restricted and that one is very deeply taking its huge negative effect on me personally…”	E6	
	“…my cooking skills have improved immensely and I would never ever have had this time to be able to bake and cook and learn new things…”	E4	
Risk of infection	“…Due to my eye operation, I was in the risk-zone, so I had to be extra careful…”	E2	16
	“…Its troublesome because people in the Tricity area are not treating this as a pandemic anymore. People are openly walking without masks, congregating and life is pretty much back to “…normal…”; “…my brother, his wife, daughter and my mother received positive COVID results in the UK and quarantined themselves for 2 weeks…”; “…My father was not with them at the time living somewhere else. They had relatively minor symptoms. My brother felt some flu like symptoms for 2 days and my mother developed a strong cough and some muscle aches. Otherwise everyone else was fine but there was some immense panic amongst family because this was near the start of the pandemic. They tested themselves at the hospital (my brother lives in a secluded neighborhood–so everyone got into the car). They arrived at a special spot where they took swab samples from everyone and PCR testing was conducted to detect COVID. Two weeks later after their quarantine ended my family received notification from their respective GP’s that they were in fact COVID positive…”; “…The COVID issue has also obviously had a financial impact on me and my family, as my father lost his job…”; “…In the meantime a large majority of my friends are working doctors in Sweden, Germany, USA, UK–and were detailing stories. Two of my friends were hospitalized with COVID; 26 year old and 30 respectively. Their symptoms were pretty severe-the 30 year old was actually intubated, the other was on high oxygen airflow–or a state E2right before intubation. Fortunately both of them got through it.…”	E3	
	“…E4I first thought the media was overreacting. That was my perspective until my faE5mily and friends became the victims of it. Not being able to visit or check on them in hospitals was heartbreaking…”; “…Surprisingly, all those folks I knew who were believed to have strong immunity and rarely fell ill all succumbed to Corona. It was hard to endure and watch those who were at their peak ages of 20′s die and to worsen the situation; some died all alone with no support from family or friends. My buddy in Stockholm opened up to the sister on how painful it was to experience the disease. He was admitted for 2–3 weeks and had to be sedated all through. His fever which kept spiking had to be monitored. Due to the illness, he lost 17 kgs. Sadly, after undergoing that awful experience, he passed away after those two weeks. The dead could not be given a decent burial…”	E2	
	“…I also was very worried especially during the peak for my sister because she was working front line as a pharmacist and I was worried because she would come home from work every day and my parents would be put at risk also so they decided to divide the house to be as safe as they can because stuck abroad and not being able to help your family or not knowing each day you could wake up to a call or a text message saying that your sister has COVID or your parents was by far definitely one of the biggest fears I had purely because I couldn’t do anything if that happened and the thought of being stuck here alone and not being able to go over in case something had happened would have haunted me for the rest of my life…”	E4	
	“…the constant worry about my family’s health and the thought that, should something happen to them, I would be unable to go offer my support or be with them. I pray every day for the health of my family, as I cannot even imagine the worst happening with me being in another country, utterly unable to each them…”	E5	

**Table 2:** Extracts from the Polish Division students’ essays discussing chosen issues.

Aspect	Examples of Extracts Extract	Participant	Number of Similar Opinions
Adaptation to e-learning	“…When the e-learning started I was challenged with installation all of necessary applications, and getting know them better It was very difficult at the beginning as I am not a person who deals well with all those platforms and IT systems.”; “…the biggest advantage is that I am not wasting my time on driving to the University, I can get up 15 min before the classes start and I am not late.	P1	20
	“…Tests on the e-learning platform were a kind of a challenge. On one side they were a motivation for a regular learning, but on the other hand they had one disadvantage, technology is not unreliable…”; “…the lack of the contact with the patient for almost half a year (from march till October) for sure can’t be omitted. Due to that I am much more scared of the beginning of my postgraduate internship…”	P2	
	“…It will be more stressful for me to take all the final exam in on-line system due to the possible technical problems that may occur…”; “…even the best lecture can’t take place of practical classes and learning on my own mistakes and improving my skills.”	P3	
	“…now instead of being in a hurry and scared of being late for the classes, wasting my time on travelling and fighting for the seat place in overcrowded classes. I can sit in my training suit with the cap of hot tea…”;”definitely I’d rather go out of that comfort zone to treat patients and get the knowledge out of the practical classes…”	P4	
	“…unfortunately, even the best lecture can’t take place of treating the real patient…”;”It is a great advantage that I am not wasting my time on driving to the University what normally took me 40 min…”	P5	
Closure of the borders	“…my father works abroad and he can’t come back home. We didn’t realize that the lockdown would last so long. If my dad came back home we wouldn’t have enough money for living, unfortunately. The most difficult was the Easter time, of course we were keeping in touch with him…”	P1	3
Social distancing	“…Before the pandemic I had regular meetings with my friends, I was going to the gym and swimming-pool etc.”, “…I have been already missing my normal, busy life, meeting friends, doing shopping…”; “…I spend my free time on cooking (I finally made my own bread), we motivated each other with my mum and did some gardening, cleaned in the garage…”; “…I was very affected by the fact that I have to stay at home…”	P1	11
	“…strengthening ties with my family, with whom I was actually made to spent time. That how it was in my case. I had some free time to do things I normally did not have time for when the life is concentrated on the University…”	P2	
	“…the possibility of getting known yourself better, new hobbies I did not have time for before. Due to the pandemic I tried new things I hope to continue when everything will come back to normality…”	P5	
Risk of infection	“…I was scared of my relatives health as many of them are older and suffering from general diseases. Thanks God they are all in good health so far…”	P2	20
	“…My grandmother is 78 years old, in good shape but.. that virus can attack anybody. My mum is a teacher and has a home office, but dad? He is going out to work every day. What if he is infected, passes the virus on me and then I will go to my grandmother with some shopping and infect her? I cannot let that happen even though have no influence on that…”	P5	

## Data Availability

The data presented in the study are available on request form the corresponding author.
